# Assessing the Role of Follow-Up Investigations After Acute Diverticulitis in Detecting Colorectal Cancer

**DOI:** 10.7759/cureus.84409

**Published:** 2025-05-19

**Authors:** Maria Borg, Emma Vella, Svetlana D Brincat, Christian Camenzuli

**Affiliations:** 1 Family Medicine, Primary Health Care, Floriana, MLT; 2 General Surgery, Mater Dei Hospital, Msida, MLT

**Keywords:** colorectal cancer, complicated diverticulitis, diverticulitis, endoscopy, follow-up

## Abstract

Background

Diverticula are small, sac-like protrusions that form along the gastrointestinal tract, most often in the sigmoid colon. While some individuals with diverticula remain asymptomatic, some patients experience symptoms, and a minority of patients go on to develop acute diverticulitis. The latter occurs when a fecalith becomes trapped within a diverticulum, leading to colonic wall damage, swelling, bacterial growth, and inflammation. A computed tomography (CT) scan is considered the gold standard imaging technique for diagnosing acute diverticulitis. However, the imaging characteristics of diverticulitis can closely resemble those of colorectal cancer (CRC), potentially obscuring a cancer diagnosis.

Aim

This audit aims to assess whether patients admitted to Mater Dei Hospital, Malta, who were diagnosed with acute diverticulitis in 2021 and 2022, adhered to the recommendations outlined in the guidelines. Furthermore, the audit will investigate the percentage of these patients who were subsequently diagnosed with CRC following a radiologically confirmed episode of acute diverticulitis.

Method

All patients with CT-confirmed diverticulitis at Mater Dei Hospital from January 2021 to December 2022 were included in this audit. Data on follow-up investigations-via imaging or endoscopy within one year of hospital presentation-were gathered from radiology reports and endoscopy records. Results were compiled and analysed using Microsoft Excel^®^ (Microsoft Corp., Redmond, WA, US).

Results

From January 2021 to December 2022, 395 patients were diagnosed with acute diverticulitis at Mater Dei Hospital, comprising 222 (56.2%) men and 173 (43.8%) women, with a mean age of 60 years. Follow-up investigations within one year were conducted in 201 (50.9%) patients. Among these, eight patients (2%) comprising five women and three men, with a mean age of 67 years, were diagnosed with CRC during follow-up. Of these, two had initially been diagnosed with uncomplicated diverticulitis, while six presented with complicated diverticulitis, characterised by perforation or abscess formation on their initial CT scans.

Conclusion

Guideline-recommended follow-up within one year of diagnosis was completed in only about half of the patients, with approximately eight (2%) patients subsequently diagnosed with CRC. It is worth noting that some patients may have undergone investigations beyond the one-year mark. Routine follow-up imaging and colonoscopy may not be necessary in every case; rather, targeted follow-up may be more appropriate for patients with complicated cases or ambiguous findings on the initial imaging.

## Introduction

Diverticula are sac-like protrusions that commonly develop along the gastrointestinal tract, particularly in the sigmoid colon. Key risk factors for diverticulosis include a low-fibre, high-fat diet, smoking, and obesity. Diverticulosis affects approximately 60% of individuals over the age of 60. Although diverticula are often asymptomatic, about 25% of individuals experience symptoms, with 5% presenting with diverticulitis [1].

Acute diverticulitis is an inflammatory condition of the diverticula, usually resulting from micro- or macro-perforations. This inflammation occurs when a fecalith becomes trapped, leading to colonic wall oedema, damage, bacterial growth, and subsequent localised inflammation and necrosis [2,3]. Certain medications, such as non-steroidal anti-inflammatory drugs (NSAIDs), steroids, and opioids, are also linked to an increased risk of diverticulitis.

The clinical presentation of diverticulitis varies significantly depending on the severity of the condition. Patients commonly experience intermittent or constant left lower quadrant pain in Western populations, whereas right lower quadrant pain is more frequently reported in Eastern Asian populations [4]. Additional symptoms may include altered bowel habits, lower urinary tract symptoms, nausea, vomiting, and fever-especially if the diverticulitis is complicated [2,3]. In more severe or "complicated" cases, patients may develop abscesses, fistulae, bowel obstruction, or perforation, all of which carry significant morbidity and are potentially life-threatening [1].

Diverticulitis is broadly classified as uncomplicated or complicated. Uncomplicated cases often respond well to conservative management. However, complicated diverticulitis presents a significant clinical burden, requiring more intensive interventions and increasing the risk of adverse outcomes. In recent years, a growing preference for non-surgical management of uncomplicated cases has emerged, supported by high-resolution diagnostic imaging-particularly contrast-enhanced computed tomography (CT) [5].

Despite its diagnostic accuracy, CT imaging presents a significant clinical dilemma: the radiological features of acute diverticulitis often mimic those of colorectal cancer (CRC). Wall thickening, fat stranding, and pericolic changes may be indistinguishable from malignancy, leading to potential delays in cancer diagnosis if follow-up is not pursued. This overlap raises a crucial question in diverticulitis management-how to balance the avoidance of unnecessary invasive investigations with the imperative to detect and treat occult malignancy.

To effectively rule out the presence of malignancy, the Royal College of Surgeons (RCS) Commission guidelines recommended that “all patients require investigation of the colonic lumen through endoscopy, barium enema, or CT-virtual colonoscopy” after the resolution of an acute diverticulitis episode [3]. This follow-up is crucial for ensuring that any hidden cancerous lesions are identified and addressed promptly, thereby improving patient outcomes and reducing the risk of late-stage diagnosis.

Aims

This audit aims to evaluate whether patients admitted to Mater Dei Hospital with acute diverticulitis had follow-up investigations within one year of presentation, as stated by the RCS Commission guidelines. In addition to assessing compliance with these guidelines, the audit seeks to determine the local incidence rate of patients diagnosed with CRC following a radiologically confirmed episode of acute diverticulitis.

## Materials and methods

All patients with CT-confirmed diverticulitis admitted to Mater Dei Hospital, the only national hospital in Malta, between January 2021 and December 2022 were included. The presence of diverticular disease was identified using a diagnosis-specific coding system utilised by the hospital. Data collected for each patient including patient demographics were extracted from clinical and laboratory records. Follow-up investigations, including radiological and endoscopy reports conducted within one year of their initial presentation, were collected and thoroughly analysed using Microsoft Excel^®^ (Microsoft Corp., Redmond, WA, US).

Study design

The presence of diverticular disease was identified using a diagnosis-specific coding admission system utilised by the hospital. Any patient with CT-confirmed diverticular disease was included in the study, alongside demographic data, from clinical and laboratory records. Follow-up investigations, including radiology and endoscopy reports conducted within one year of the initial presentation, were collected and inputted into Microsoft Excel^®^. Data were organised accordingly and analysed statistically.

Sample size

Three hundred ninety-five individuals satisfied the inclusion criteria and, hence, were in the sample between January 2021 and December 2022.

Inclusion Criteria

Only patients with CT-confirmed acute diverticulitis were included in the study.

Exclusion Criteria

Suspected diverticular bleeding, unconfirmed CT results, or incidental diverticular disease discovered during colonoscopy were excluded.

Ethics statement

Ethical clearance was obtained from the Hospital's Ethical Board. Audit approval was obtained from the Hospital's Data Protection Board, the Hospital's CEO, and the Chairman of the Surgery Department.

## Results

Between January 2021 and December 2022, a total of 395 patients were admitted with a diagnosis of acute diverticulitis. Among these patients, 222 (56.2%) patients were male and 173 (43.8%) patients were female (Table 1).

**Table 1:** Distribution of sex and follow-up rates across total, uncomplicated, and complicated cases

	Total (N = 395)	Uncomplicated (N = 243)	Complicated (N =152)
Male	222 (56.2%)	116 (49.5%)	106 (50.5%)
Female	173 (43.8%)	115 (58.9%)	72 (41.0%)
Follow-up performed	201 (50.9%)	101 (50.2%)	100 (49.8%)

**Figure 1:**
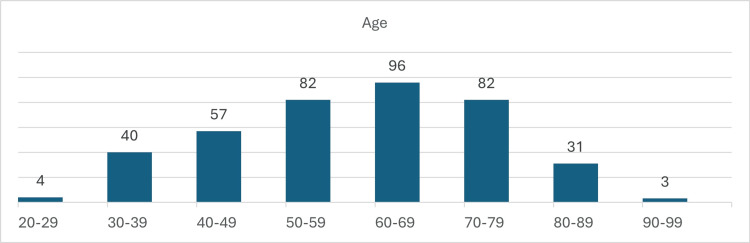
Bar chart showing the mean age of diagnosis

The majority of patients, specifically 96 individuals, fell within the 60-69 age range, followed by 82 patients aged 50-59 and 70 patients aged 70-79. Notably, four patients were younger than 30 years, as per Figure 1 and Table 2.

**Table 2:** Comparison of age groups between uncomplicated and complicated presentations

Age group (years)	Total (N = 395)	Uncomplicated (N = 243)	Complicated (N = 152)
<30	4	4	0
30–39	40	23	17
40–49	57	32	25
50–59	82	48	34
60–69	96	57	39
70–79	82	56	26
80 and above	34	23	11
Total	395	243	152

**Figure 2:**
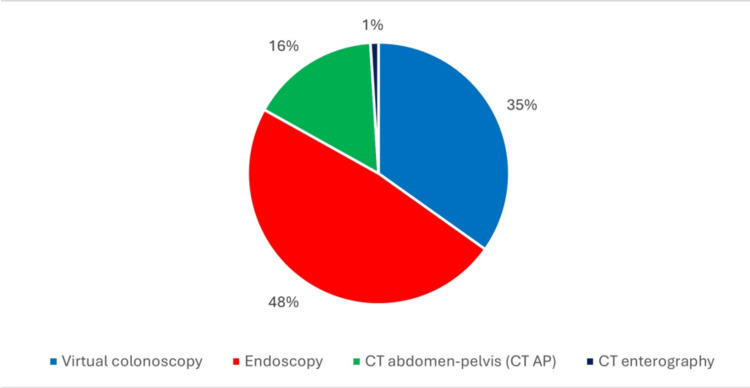
Pie chart showing the method of follow-up investigation CT: computed tomography

Follow-up investigations were conducted for 201 patients, indicating that just 50.9% of the total cohort underwent further evaluation within one year of their diverticulitis diagnosis and resolution. The most common form of follow-up imaging was endoscopy, utilised in 97 (48%) patients, followed by CT-virtual colonoscopy in 70 (35%) patients, CT abdomen and pelvis in 32 (16%) patients, and CT enterography in two (1%) patients, as seen in Figure 2.

Among the 201 patients who received follow-up, CRC was identified in eight individuals, confirmed through histopathological analysis. Of these patients, five were female and three were male, with a mean age of 67 years. Notably, only two individuals had uncomplicated diverticulitis, while the remaining six were diagnosed with complicated diverticulitis due to perforation or abscess formation as indicated on the initial CT scan.

## Discussion

This audit provides valuable insights into follow-up care following acute diverticulitis, analysing data from 395 patients. Notably, only 201 (50.9%) patients underwent follow-up evaluations of the colonic lumen, resulting in a CRC detection rate of 2%, which corresponds to eight cases. In each of these eight cases, the diagnosis of CRC was confirmed histologically as adenocarcinoma. Among these patients, three were diagnosed with adenocarcinoma after undergoing surgical procedures for what was initially presumed to be diverticulitis, with the diagnosis being established through histopathological analysis of the excised specimens. The CRC detection rate observed in this audit was found to be comparable to findings from other medical centres that have conducted similar audits. For example, St. Paul’s Hospital in Vancouver, Canada, reported a detection rate of 2.9% [6], while Sandwell and West Birmingham Hospitals NHS Trust in the UK documented a rate of 2.1% [7].

The low follow-up rate observed among diverticulitis patients presents both clinical and public health concerns. Timely follow-up is essential for monitoring complications, assessing response to treatment, and ensuring appropriate long-term management, especially in cases classified as complicated. Inadequate follow-up may result in missed opportunities to identify recurrent episodes, the progression to chronic complications (e.g., strictures and fistulas), or even underlying malignancy mimicking diverticulitis, particularly in older populations.

From a systems perspective, poor follow-up can compromise the continuity of care and undermine efforts to standardise best practices in diverticular disease management. It may also lead to increased healthcare utilisation in the form of emergency department revisits or unplanned hospital readmissions, escalating costs, and patient morbidity.

Multiple international guidelines recommend a follow-up colonoscopy to examine for CRC in all patients following an episode of acute diverticulitis. This is the position taken by the RCS of England, the Association of Coloproctology of Great Britain and Ireland, and the American Society of Colon and Rectal Surgeons [8]. However, emerging data are providing new insights into the management, particularly the follow-up of uncomplicated cases of diverticulitis. A recent UK-based study investigated patients over the age of 18 with CT-confirmed uncomplicated left-sided diverticulitis between 2014 and 2017. In this study, follow-up colonoscopies were performed within four to six weeks of the initial diagnosis. The findings revealed that there was virtually no diagnostic gain from these imaging procedures, as no cases of CRC or advanced adenomas were identified among the 204 patients included in the investigation [8]. Similarly, in our study, only two patients (0.5% of the total cohort) with uncomplicated diverticulitis were diagnosed with CRC, while the majority had complicated cases involving an abscess or perforation. These results raise important questions regarding the necessity of follow-up imaging for uncomplicated diverticulitis cases. As the pressure on medical resources continues to grow, it is essential to evaluate both the economic value and clinical benefits of follow-up endoscopy after uncomplicated diverticulitis. Given that CT imaging exhibits a sensitivity of 94% and a specificity of 99%, the benefit of additional follow-up colonoscopy and CT scans for uncomplicated cases becomes increasingly questionable [9]. Thus, these findings suggest a need for a re-evaluation of the protocols surrounding follow-up imaging in cases of uncomplicated diverticulitis, emphasising the importance of an individualised patient assessment to determine the most appropriate course of action.

The recommendations stemming from this audit emphasise the critical need to enhance awareness regarding the importance of timely follow-up within one year after a diagnosis of CT-proven diverticulitis, particularly in complicated cases. To effectively convey this message, the implementation of educational posters in surgical wards and outpatient clinics can serve as a valuable tool. These visual aids can remind both healthcare providers and patients about the necessity of follow-up investigations. Additionally, a closed-loop audit will be performed locally in the coming year to evaluate whether the adherence rates to the RCSEng Commission guidelines improve following the implementation of recommendations. The closed-loop audit will also indirectly evaluate the impact of the COVID-19 pandemic on follow-up rates and its potential effect on the detection rate of CRC. By systematically reviewing follow-up practices over time, we can gain insights into trends, identify areas requiring improvement, and ultimately ensure that patients receive the appropriate care they need after an episode of diverticulitis, especially if it has been a complicated one.

It is essential to acknowledge the limitations inherent in this audit. First and foremost, the data collected did not allow for an accurate estimation of the proportion of patients who may have been unfit to undergo colonoscopy due to underlying medical conditions. Additionally, the COVID-19 pandemic, which began in 2020, likely contributed to a significant backlog of follow-up investigations, leading to delays in assessments that extended beyond the one-year mark. Furthermore, this audit did not account for imaging studies and colonoscopies performed in private healthcare settings, despite the fact that some patients may have initially received their diagnoses at the national hospital. This oversight limits the comprehensiveness of the follow-up data.

Moreover, while some patients may have been offered follow-up investigations, it is possible that they declined these opportunities, and such refusals were not tracked in this audit. It is also worth noting that several patients exhibited varying degrees of dysplasia; however, these findings were not included in the analysis, as the focus was solely on confirmed cases of colorectal malignancy. These limitations highlight the need for caution when interpreting the results of this audit and suggest areas for improvement in future studies. Addressing these limitations will enhance the accuracy and reliability of findings related to follow-up practices for acute diverticulitis and associated malignancies.

## Conclusions

This audit indicated that only 201 (50.9%) patients received a colonic lumen follow-up within the first year of their diverticulitis diagnosis. The incidence of CRC in this cohort was detected in eight (2%) patients, with the majority of cases occurring in patients presenting with complicated diverticulitis. These findings underscore the need to carefully evaluate the justification for invasive and uncomfortable procedures, such as follow-up colonoscopies, which carry associated risks. Consequently, future research should investigate the diagnostic value of colonoscopy following uncomplicated left-sided acute diverticulitis to assess whether its routine use is warranted for this patient group.
